# Issues Concerning the Mechanisms of Bone Conduction

**DOI:** 10.3390/audiolres14050070

**Published:** 2024-09-17

**Authors:** Miriam Geal-Dor, Cahtia Adelman, Haim Sohmer

**Affiliations:** 1 Speech & Hearing Center, Hebrew University-Hadassah Medical Center, Jerusalem 91200, Israel; cahtiaa@hadassah.org.il; 2 Department of Communication Disorders, Hadassah Academic College, Jerusalem 91200, Israel; 3 Department of Medical Neurobiology (Physiology), Hebrew University-Hadassah Medical School, Jerusalem 91120, Israel; haims@ekmd.huji.ac.il

**Keywords:** bone conduction, mechanisms, sensori-neural, middle ear

## Abstract

Air and bone conduction thresholds are used to differentiate between conductive and sensori-neural hearing losses because bone conduction is thought to bypass the conductive apparatus, directly activating the inner ear. However, the suggested bone conduction mechanisms involve the outer and middle ears. Also, normal bone conduction thresholds have been reported in cases of lesions to the conduction pathway. Therefore, further investigation of bone conduction mechanisms is required.

## 1. Differentiation Between a Sensori-Neural and a Conductive Hearing Loss

The determination of the thresholds to air conduction (AC) and bone conduction (BC) stimuli is used in the clinic to differentiate between a sensori-neural hearing loss (SNHL, in which both AC and BC thresholds are elevated) and a conductive hearing loss (CHL, in which AC thresholds are elevated, with normal BC thresholds). The BC mechanisms are considered to be challenging and not well understood [1,2,3]. Therefore, in order to properly evaluate these threshold determinations, especially the BC thresholds, it is imperative to adequately understand the mechanisms leading to the responses elicited by the BC stimulation. On the one hand, the threshold to BC stimulation is thought to contribute to a differential diagnosis between a sensori-neural hearing loss and a conductive hearing loss because, as stated: BC stimulation effectively “short-circuits the middle ear” [4], or “the bone conduction signal bypasses the outer and middle ears and directly stimulates the cochlea” [5], and “excite the inner ear and cochlea, directly bypassing the outer and the middle ears” [6] or it is “believed to reflect the true cochlear function” [3], or “the pathway considered to be the primary one is altogether osseous from the first skull bone to the cochlear capsule” [7]. Taken together, these authors suggest that BC responses contribute to diagnoses between a CHL and SNHL because they apparently bypass the conduction pathway.

## 2. Mechanisms of BC

The resulting skull vibrations are conducted through the skull bone (therefore called “bone conduction”) to the ear. The mechanisms thought to be involved in bone conduction include: at the outer ear, vibrations of the wall of the external auditory canal are induced. The air pressures elicited by the vibrations of the walls of the external canal are usually dissipated in the surrounding air. However, if the opening of the canal is occluded, the air pressures trapped in the occluded canal induce vibrations of the tympanic membrane, the middle ear ossicular chain, and the stapes footplate in the inner ear, i.e., by eliciting vibrations of the conductive pathway (by an AC-like mechanism). This BC mechanism is referred to as the occlusion effect. In parallel with this outer ear BC mechanism, the bony wall of the middle ear and middle ear ossicles vibrate together. However, at some higher frequencies, the vibrations of the ossicles cannot keep up with the vibrations of the middle ear wall, eliciting the BC mechanism referred to as ossicular chain inertia. In addition, the vibrations of the bony capsule of the inner ear (the cochlea) result in the inner ear BC mechanisms of inner ear fluid inertia and the distortion (compression, expansion) of the walls of the inner ear. These inner ear BC mechanisms are dependent on the impedance of the round window, being 20 times lower than that of the oval window (due to the attachment of the ossicular chain to the oval window, while the round window is only a simple membrane). As a consequence, the resulting excursions of the round window are greater than those of the oval window, so that the basilar membrane situated between the cochlear scalae is displaced accordingly, leading to a traveling mechanical wave along the basilar membrane. Stenfelt and Goode suggest that inner ear fluid inertia is the major effective BC mechanism, especially at the lower frequencies [3]. These four BC mechanisms presumably act simultaneously with each other so that the phase relations between them can lead to mutual cancellation or mutual augmentation. In addition, the thresholds and the frequency ranges of maximal effectiveness of each of them are presently not clear. In the final analysis, the BC mechanisms activate the inner ear by producing inner ear fluid displacements between the two inner ear windows (oval window and round window) and compartments (scala vestibuli and scala tympani).

## 3. BC Mechanisms and the Conduction Pathway

As can be seen from the BC mechanisms reviewed above, most or all of them are in some way based on the normal function of the tympanic membrane, middle ear ossicles, and the relative impedances of the oval and round windows, i.e., to some extent, they require the normal function of the conductive pathway or apparatus. For example, the occlusion effect requires the tympanic membrane, and also the middle ear ossicles, in order to conduct the vibrations of the tympanic membrane and the ossicles to excite the inner ear. The ossicle inertia mechanism requires the presence of the middle ear ossicles. The ossicles also provide a higher impedance of the oval window, enabling the inner ear BC mechanisms. Therefore, the statement that BC responses contribute to the differential diagnosis between SNHL and CHL because BC stimulation effectively bypasses the outer and middle ears is apparently inconsistent with the BC mechanisms as described and reviewed by the workers in the field [3,4,5,6,7]. The BC mechanisms as described apparently require the normal function of the tympanic membrane and the middle ear ossicles, which are dependent upon them, thus involving the outer and middle ears.

In addition, BC thresholds in the normal range have been reported in post-radical mastoidectomy and subtotal petrosectomy patients [8,9], in whom the tympanic membrane and the middle ear ossicles had been surgically removed. In such patients, the occlusion effect of the outer ear and the middle ear ossicular inertia mechanisms would, therefore, be compromised. In addition, the removal of the ossicles would not only remove the mechanism of ossicular inertia but would also reduce the impedance acting on the oval window and thereby affect the inner ear BC mechanisms of inner ear fluid inertia and inner ear distortion. Furthermore, BC thresholds in the normal range have even been found in cases with congenital absence of the round window and of the oval window [10,11,12], i.e., the inner ear BC mechanisms were not adversely affected by the absence of the windows. Also, in cases of otosclerosis (fixation of the stapes footplate), the BC thresholds were not affected by restoring the mobility of the ossicles in the course of stapedotomy surgery [13]. Furthermore, in mice models of CHL, evoked auditory brainstem responses to BC stimulation have been reported to be insensitive to the induction of conductive pathologies (removal of the tympanic membrane, malleus, incus, or filling the middle ear cavity with saline) [14].

The previous paragraph clearly reflects the nature of an additional issue concerning the mechanisms of BC: on the one hand, a normal BC threshold coupled with an elevated AC threshold (i.e., the presence of an air–bone gap) serves as a clear sign in the clinic of a conductive hearing loss, i.e., reflecting a lesion of the conductive apparatus. However, it is apparent that BC thresholds in the normal range have nevertheless been found in cases presenting with lesions to the conductive pathway [8,9,10,11,12,13,14], including absence and an abnormal function of vital components, from the tympanic membrane to the middle ear ossicles to the two inner ear windows: the round window and the oval window. Perhaps an alternative BC mechanism is effective in such conditions.

There are thus two issues concerning the mechanisms of BC, which, together, lead to the conclusion that further study is required to evaluate the mechanisms of BC:1: the statement that BC responses effectively bypass the conduction pathway is inconsistent with the mechanisms of BC, as described, which require a normal conductive apparatus; 2: the finding that normal BC thresholds have been reported, in spite of lesions to the conductive pathway, should lead to the search for an alternative explanation of the BC mechanisms that can truly bypass the pathway. Testing of the BC threshold in the clinic for diagnosis is useful and will continue, but there is a need to further investigate the underlying BC mechanisms that might better explain the results in the different pathologies.

These issues apparently serve as the basis for the impression that BC mechanisms are challenging and not well understood [1,2,3] and should lead to reconsideration of the BC mechanisms, as described. In summary, the mechanisms of BC are truly complicated and, apparently, not sufficiently clear. In conclusion, these issues should lead to the planning and conduction of experiments designed to provide clues as to the mechanisms involved in the activation of the inner ear in the presence of lesions to the conductive pathway, thus contributing to the resolution of the issues. For example, since the fluid pressures within the cranial cavity may be affected by BC stimulation, designing an experiment with possible activation of the inner ear through soft tissue fluid communications between the fluid contents of the cranial cavity and the inner ear fluids should be assessed. In addition, it may be helpful to carefully analyze the effects of stimulus frequency on air and bone conduction thresholds, especially the BC thresholds activated by each of the suggested BC mechanisms.

## Data Availability

Not applicable.
